# Study of MRI brain findings and carotid US features in systemic sclerosis patients, relationship with disease parameters

**DOI:** 10.1186/s13075-019-1877-z

**Published:** 2019-04-15

**Authors:** Rania M. Gamal, Hanan Sayed M. Abozaid, Mohmed Zidan, Mohamed Aboel-Kassem F. Abdelmegid, Mohmed Raouf Abdel-Razek, Sahar Abdel-Rahman Alsayed, Amr F. Mourad, Nashwa Mostafa A. Azoz, Lobna Abdel-Wahid Mohram, Daniel E. Furst

**Affiliations:** 1 Rheumatology and Rehabilitation Department, Assiut University Hospital, Assiut, 71515 Egypt; 2 Rheumatology and Rehabilitation Department, Sohag University Hospital, Sohag university, Sohag, 82524 Egypt; 3 Diagnostic Radiology Department, Assiut University Hospital, Assiut, Egypt; 4 Cardiovascular Medicine Department, Assiut University Heart Hospital, Assiut, Egypt; 5 Diagnostic Radiology Department, South Egypt Cancer Institute, Assiut, Egypt; 6 Internal Medicine Department, Assiut University Hospital, Assiut, Egypt; 7 Division of Rheumatology, University of California in Los Angeles (Emeritus), Los Angeles, USA; 8 Department of Medicine, University of Washington, Seattle, WA USA; 9 Division of Rheumatology and Experimental Medicine, University of Florence, Florence, Italy

**Keywords:** Systemic sclerosis, Intima-media thickness, Brain MRI

## Abstract

**Background/objectives:**

Systemic sclerosis (SSc) is an autoimmune disease associated with immune abnormalities and widespread vascular lesions, including increased intimal and medial thickness. These changes may be reflected in early atherosclerosis and cardiovascular risks. We aimed in this study to examine the carotid artery intima-media thickness and MRI brain findings in SSc patients and compared them to a group of normal controls. A relationship between these parameters and clinical measures in SSc was also sought.

**Methods:**

Seventy-two SSc patients with no central nervous system (CNS) symptoms and 42 healthy controls were included. Clinical and laboratory measures, Medsger’s severity scale, and Doppler ultrasound common carotid artery intima-media thickness (CCA-IMT) were measured. Brain fluid-attenuated inversion recovery (FLAIR)-MRI and diffusion-weighted MRI (DWI) were also done.

**Results:**

SSc patients had more CCA-IMT, higher CRP, and more brain MRI hyperintense lesions than controls (*P* < 0.05). Significant positive correlations existed between CCA-IMT and Medsger vascular (*r* = 0.7, *P* = 0.02). The FLAIR-MRI showed multiple hyperintense lesions in 24 patients (33%), ranging 0–36 lesions. SSc patients with more lesions (positive MRI) had longer disease duration (*P* = 0.001) and left and right carotid artery atheromata (*P* = 0.001, and 0.013, respectively) than SSc patients with negative MRIs; Medsger vascular score did not separate the SSc groups (*P* = 0.08).

**Conclusions:**

In systemic sclerosis patients without central nervous system symptoms, MRI lesion numbers correlated with CCA-IMT. MRI abnormalities were found more frequently if CRP was elevated, if the Medsger SSc Severity Scale was increased, or if there was thickened carotid IMT.

## Introduction

Systemic sclerosis (SSc) is a connective tissue disorder with widespread vascular lesions, possibly explained by immune reactions to viral or environmental factors, reperfusion injury, or anti-endothelial antibodies [1, 2] accompanied by angiogenesis which is insufficient or defective [3, 4]. Most studies have been directed to microvascular disease in SSc, but some studies have suggested an increased prevalence of macrovascular disease as well [5, 6]. The development of accelerated atherosclerosis in SSc is less clear, although, an increase in carotid intima-media thickness (IMT) in SSc patients has been reported [7, 8]. Clinically there is little evidence for increased macrovascular complications such as stroke or myocardial infarction [5].

High-resolution ultrasound is a non-invasive and practical method to examine peripheral arteries and carotid lesions, including increased intima-media thickness and atherosclerotic plaques [9]. The studies of the IMT in Egyptian patients with other rheumatic diseases documented subclinical atherosclerosis in rheumatoid arthritis [10], systemic lupus erythematosus [11], and primary osteoarthritis [12].

Some studies have concluded that ultrasound-detected carotid atherosclerotic lesions are associated with ischemic stroke [13, 14] and can help estimate the risk of ischemic stroke [15–17] Accordingly, both the Japanese Society of Hypertension Guidelines for the Management of Hypertension and the European Society of Hypertension/European Society of Cardiology 2003 Guidelines for the Management of arterial hypertension have added increased CAA-IMT and common carotid artery plaques (CCA) as risk factors for stroke [18, 19].Per the Japanese Society for the Detection of Asymptomatic Brain Diseases, a silent cerebral infarct (SCI) must be ≤ 3 mm in greatest diameter [18]. The SCI can be detected by magnetic resonance imaging (MRI) and is considered an important risk factor for stroke [20, 21] and is associated with both psychiatric and neurologic disorders [22, 23].

Diffusion-weighted MRI (DWI) provides in vivo pathological information and allows the differentiation of acute stroke from chronic stroke or from non-specific white matter lesions [24]. Fluid-attenuated inversion recovery (FLAIR)-MRI demonstrated that it can detect brain abnormalities in many neurological diseases with particularly high sensitivity to detect lesions in the periventricular and subcortical regions [25].

Based on these data, we wished to examine the thickness of the carotid artery intima-media and the brain MRI-detected lesions in systemic sclerosis patients to compare them to age- and sex-matched normal controls. Secondary analyses examined large vessel disease in the SSc patients and sought to find any relationships of the carotid and CNS MRI findings with SSc clinical disease and selected laboratory parameters.

## Methods

Systemic sclerosis patients at the outpatient clinics of Rheumatology & Rehabilitation Department in Assiut University Hospital were invited to participate in this study. All patients and volunteers signed voluntary informed consent. The research was approved by the clinical research ethics committee of Assiut University Hospitals NO 17300270. The inclusion criteria for the study were SSc by the 2013 American College of Rheumatology (ACR) classification criteria for scleroderma [26], with either diffuse (dcSSc) or limited cutaneous (lcSSc) disease (Le Roy et al) [27], age at disease onset after 16 years old, and stable medication in the last 3 months if medications were used. Forty-two healthy volunteers (38 female and 4 male), recruited from among university and hospital workers, served as age- and sex-matched controls,

The exclusion criteria were (1) mixed connective tissue disease and/or other autoimmune connective tissue pathologies overlapping with SSc; (2) systemic diseases known to involve the central nervous system CNS (e.g., renal ( not including scleroderma renal crisis) and liver diseases, AIDS); (3) chronic medical illness associated with atherosclerosis such as diabetes mellitus or hypertension; (4) history of psychosis or other neurological diseases; (5) use of drugs (other than SSc treatments) known to cause neurotoxicity such as NSAID, anti-coagulants, anti-HBP drugs, or hypoglycemic; (6) cancer within the previous 5 years; or (7) drug abuse or alcoholism.

All selected eligible participants were assessed clinically, and the following data were obtained. (1) Demographic data, smoking status, and history and clinical examination, both general and neurological, were recorded. For SSc patients, disease duration, articular and extra-articular organ involvements, signs of skin involvement, and treatment regimens were also recorded. (2) Routine laboratory investigations including erythrocyte sedimentation rate (ESR), C-reactive protein (CRP), lipid profile (total cholesterol, high-density lipoprotein (HDL), low-density lipoprotein (LDL), triglycerides (TG)), and random blood sugar (RBS). (3) Routine assessment of the disease: (i) duration, frequency, and vasodilators treatment of Raynaud’s disease if required; presence of digital pitting scars, digital tip ulcers, or gangrene; (ii) modified Rodnan skin score (mRSS) [28, 29]; (iii) fingertip-to-palm (FTP) distance in flexion; (iv) proximal muscle power assessment; (v) gastro-intestinal tract manifestations; (vi) estimated pulmonary artery systolic pressure (PASP) by Doppler echo; (vii) forced vital capacity (FVC), % predicted; (viii) electrocardiogram (EKG); (ix) left ventricular ejection fraction (LVEF) by echo Doppler; (x) history or presence of scleroderma renal crisis (SRC) and serum creatinine measurement. (4) Revised Medsger’s SSc severity scale [30].

Carotid Doppler ultrasound examination utilized a Philips HDI 5000 duplex with a 7.5–12-MHz linear array. The CCA-IMT was measured 1 cm distal to the carotid bifurcation in the posterior wall. For each patient, the highest CCA-IMT among the measured segments studied on each side was recorded. Per current sonographic criteria, CCA-IMT was considered “normal” when < 0.9 mm, “thickened” when CCA-IMT ≥ 0.9 mm, and indicative of atherosclerotic plaque when the thickness was > 1.3 mm [31].

All participants underwent radiologic evaluation of the extent and localization of brain abnormalities using conventional MRI and DWI of the brain. Conventional MRI was performed using 1.5 T (Achieva, Philips, Netherlands). After centering with a three-step scout (axial, coronal, and sagittal), an axial spin echo, FLAIR-weighted sequence was acquired with the following parameters: time of repetition/time of echo = 11000/140, matrix 232/512, and field of view 230 mm with thickness of 5 mm (1-mm gap), for a total of 23 images with NSA 2. The FLAIR images were used for counting focal brain abnormalities in both SSc patients and controls. The FLAIR is more sensitive for periventricular lesions in white matter than other weighted images. Diffusion-weighted sequence was performed as an echo-planar sequence with time of repetition/time of echo = 3773/117, matrix 152/256, and field of view 230 mm with thickness of 5 mm (1-mm gap), for a total of 23 images with NSA 1 and EPI factor 55. The DW images were acquired in 0 and 1000 s/mm^2^ cuts and the diffusion gradients applied simultaneously in three orthogonal spatial directions. These images were evaluated by a radiologist who counted the white matter hyperintensities in all brain regions. All the abnormalities were electronically measured (along the longest diameter) to distinguish the lesions into three groups: < 2 mm, ≥ 2 to < 5 mm, and ≥ 5 mm

### Statistical analysis

Data were analyzed using SPSS version 20.0 statistical package. Data were presented as number and percent, mean ± SD, or median and range as appropriate. Student’s *t* test and univariable and multivariable analyses were used to compare the SSc patients to our age- and sex-matched group of normal controls. We also examined differences within the SSc patient groups, comparing limited to diffuse cutaneous disease.

Pearson correlation coefficients (*r*) were used to measure the associations among quantitative variables, examining the carotid IMT measures and MRI lesions and sizes with clinical and laboratory measures in the SSc patients. *P* value < 0.05 was considered statistically significant. No compensation was made for repeated analyses.

LOCF accounted for missing data. We found no significant difference compared to the completer-only analysis, so only the completer analysis is presented.

## Results

Seventy-two SSc patients (65 females (90%) and 7 males) were compared to 42 age- and sex-matched healthy controls (38 females (90%) and 4 males). The controls were recruited from the hospital offices, clinics, and laboratories, matched for the age and gender with the SSc patients. They had no history of connective tissue, cardiac, or central nervous system diseases and did not take any medications other than vitamins. All study participants, both normal and SSc patients, were nonsmokers.

Tables 1 and 2 demonstrate the clinical and demographic data for SSc patients and controls. Among the SSc patients, disease duration was 6.9 ± 5.6 years. Seventy-nine percent were ANA positive, 64% were anti-SCL70 positive, and 29% were anti-centromere positive. Forty-seven patients (65%) had limited SSc while 25 (35%) had diffuse SSc. Fifty-seven (75%) patients had Raynaud’s phenomena, 23 (31.9%) had digital ulcers, 22 (30.6%) had telangiectasia, and 35 (79.2%) had calcinosis. Modified Rodnan skin score (MRSS) was 24.9 ± 9.3; high-resolution CT of the lungs (HRCT) showed fibrosis in 33% of the patients; 19 (26.4%) had SRC.

**Table 1:** Clinical and selected laboratory findings among the SSc and control groups

	SSc group *N* = 72	Control group *N* = 42	*P* values
SSc disease duration years (mean ± SD)	6.9 ± 5.6		
ANA	79%		
Anti- SCL70	64%		
Anti-centromere	29%		
Raynaud’s phenomena	75%		
Digital ulcers	31.9%		
Dyspnea and heart burn	72.2%		
MRSS	24.9 ± 9.3		
ECG finding, normal	45 (62.5%)	42 (100%)	0.009
Rt CCA atheroma	30 (41.7%)	0 (0%)	0.001
Lt CCA atheroma	30 (41.7%)	0 (0%)	0.001
Cr. clearance, normal	66 (91.7%)	42(100%)	0.150
CRP; normal	24 (33.3%)	42(100%)	0.000
Family history of CV disease, negative	46 (64%)	30 (71.4%)	0.405
Brain diffusion MRI, positive	0 (0%)	0 (0%)	
Brain FLAIR-MRI, hyperintense lesions	25 (34.7%)	0 (0%)	0.004

*P* values < 0.05 are significant

**Table 2:** Medsger severity scales (*N*)

Organ system	Normal 0	Mild 1	Moderate 2	Severe 3	End stage 4
General	0	23	12	12	25
Peripheral vascular	15	14	24	20	3
skin	0	20	17	8	27
Joints/tendons	3	32	9	16	12
Muscles	12	30	18	12	0
GIT	17	13	30	20	2
Lungs	3	40	12	17	0
Heart	23	19	22	8	0
Kidney	53	3	6	8	2

Table 3 compares the laboratory and risk factors with respect to atherosclerosis, comparing SSc patients to controls. There were statistically significant differences between the patients and the controls in each of RT-CCA-IMT, LT-CCA-IMT, CRP, ESR, triglycerides, cholesterol, and high-density lipoproteins (HDL). There were no significant differences between patients and controls for other routine values, such as Hgb, WBC, and creatinine levels.

**Table 3:** Potential risk factors for atherosclerosis—SSc vs control

	SSc cases Mean ± SD	Control Mean ± SD	*P* value
BMI	24.9 ± 6.5	26.3 ± 6.5	0.631
Systolic blood pressure	122.5 ± 7	121.8 ± 7.7	0.814
Diastolic blood pressure	79.3 ± 8.7	79.3 ± 7.4	0.891
Rt CCA IMT	0.96 ± 0.5	0.6 ± 0.1	0.001
Lt CCA IMT	0.9 ± 0.4	0.6 ± 0.1	0.003
% of positive CRP	48 (66.7%)	0(0%)	0.001
ESR	26.6 ± 19.4	7.2 ± 2.1	0.001
HB	11.9 ± 1.1	12 ± 1.2	0.716
Blood urea nitrogen	4.3 ± 1.8	4.6 ± 1.2	0.176
Serum creatinine	57.9 ± 21.8	57.9 ± 12.5	0.351
Blood sugar	5.3 ± 1.3	4.8 ± 1	0.688
Triglyceride	105.8 ± 35.1	82.9 ± 23.3	0.005
Cholesterol	164.6 ± 32.6	129.1 ± 20.9	0.001
HDL	44.1 ± 9.2	64.9 ± 9.9	0.001
LDL	104.2 ± 25.8	95.6 ± 14.8	0.216

There was no significant difference between the right and left CCA-IMT (*P* = 0.616), so we combined the CCA-IMT (Table 4).

**Table 4:** Correlation between CCA IMT and different variables: the table displays the variables which showed a significant correlation

Variables	*R*	*P* value
Medsger’s kidney	0.582	0.029
Medsger’s general	0.469	0.001
Cholesterol	0.457	0.001
HRCT	0.589	0.025
Age	0.721	0.001
Dysphagia	0.620	0.013

**Fig. 1:**
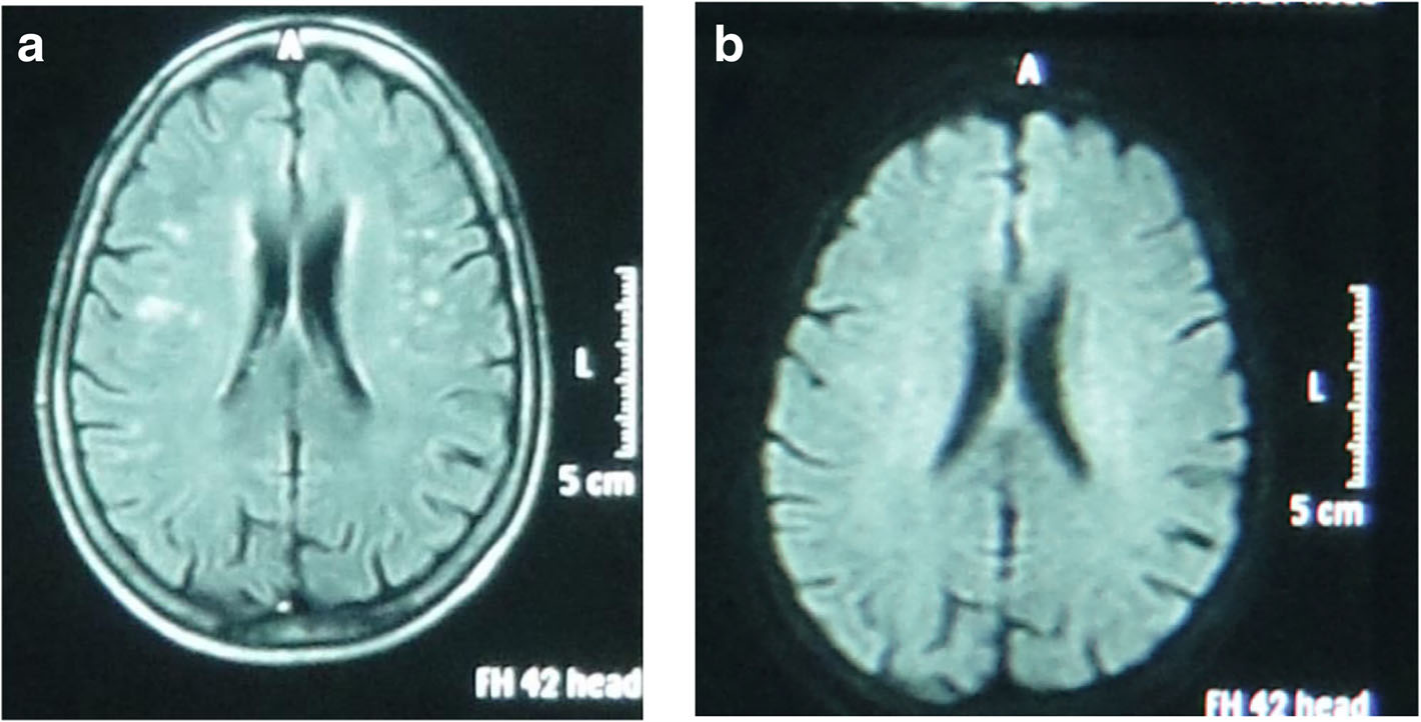
Brain MRI for a female patient aged 42 years who suffered from SSc, disease duration: 5 years. **a** FLAIR-MRI shows multiple rather well-defined hyperintense. signal foci seen in the deep periventricular white matter of both parietal regions with no comparable signal abnormality on DWI (**b**) suggesting late subacute to chronic ischemic lesions

The brain diffusion MRI did not show any abnormalities in the patient group while the brain FLAIR-MRI showed multiple hyperintense lesions in 24 SSc patients (33%) (see example in Fig. 1) versus 6 controls (14%) (*P* = 0.01).

Lesion numbers in the brain FLAIR-MRI ranged from 0 to 34 in the patients vs 0 to 7 in the controls (*P* = 0.02), and the lesions were larger among patients when the lesions were classified into 3 groups (< 2 mm, ≥ 2 mm and ≥ 5 mm) (*P* = 0.05).In univariable correlations, there were significant positive correlations of the MRI lesion numbers with the Medsger vascular scale (*r* = 0.7, *P* = 0.02), CCA-IMT (*r* = 0.6, *P* = 0.042), and LDL (*r* = 0.5, *P* = 0.03).

Among the SSc patients, there were significant positive correlations of MRI lesion size with age (*r* = 0.7, *P* = 0.01), disease duration (*r* = 0.6, *P* = 0.03), and MRSS (*r* = 0.63, *P* = 0.01) but no other variables.

Compared to MRI-negative SSc patients, those with positive MRI findings had longer disease duration (*P* = 0.001), longer Raynaud’s attacks (*P* = 0.02), more atheromatous disease (*P* = 0.001–0.013), and thicker CCA-IMT (*P* = 0.01) (Table 5).

**Table 5:** The only variables which had a significant difference in a comparison between patients with positive FLAIR-MRI findings and negative FLAIR-MRI

Variables	+ve MRI findings	−ve MRI findings	*P* value
Disease duration	11.75 ± 7.2	3.13 ± 1.6	0.001
Raynaud’s duration (minutes)	18.13 ± 9.9	8.13 ± 4.4	0.02
Rt CAA atheroma	0.25 ± 0.15	0.0 ± 0.0	0.013
Lt CAA atheroma	0.38 ± 0.18	0.0 ± 0.0	0.001
CAA-IMT	1.25 ± − .65	0.87 ± 0.18	0.01

Patients with thicker CCA-IMT (≥ 0.9 vs < 0.9 mm), thus examining large vessel disease, had higher mean Medsger kidney severity score (1.0 vs 0.0, *P* = 0.001), more MRI lesions (5.4 vs 0.9, *P* = 0.001), and larger MRI lesions (0.5–1.0 with *P* = 0.01).

**Fig. 2:**
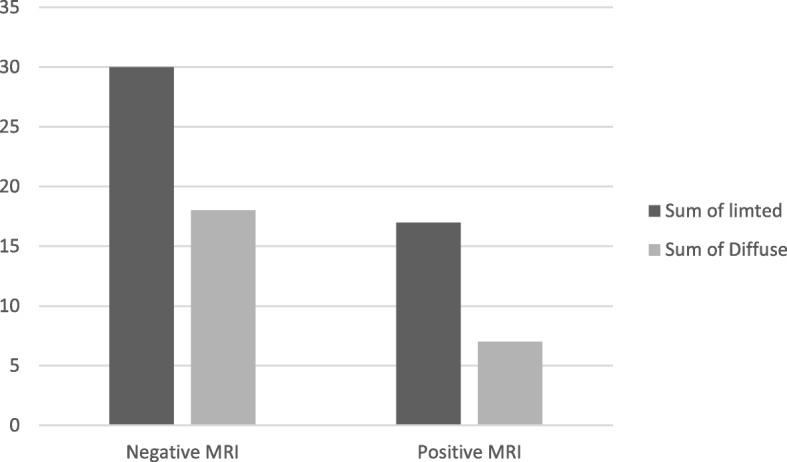
A graph compares diffuse and limited SSc patients in regard to +ve MRI brain findings. There is no significant difference between the MRI +ve results with the type of Scleroderma

There were no statistically significant differences in the frequency of MRI hyperintense lesions between limited and diffuse scleroderma patients (Fig. 2), and there were numerical but non-significant correlations between a generalized inflammatory marker (CRP), MRI lesions numbers, and atheromata in the CCA.

In multiple regression analysis, CCA-IMT (*r* = 0.8, *P* = 0.006), more severe Medsger kidney (*r* = 0.5, *P* = 0.05) and Medsger vascular scores (*r* = 0.8, *P* = 0.01), higher cholesterol (*r* = 0.7, *P* = 0.02), and higher LDL (*r* = 0.5, *P* = 0.02) were independent contributors to the presence of more MRI lesions.

## Discussion

Central nervous system involvement in patients with SSc was generally considered an uncommon feature of the disease. However, several studies have reported CNS abnormalities with descriptions of carotid and intracranial arterial abnormalities and with occasional transient ischemic attacks, ischemic stroke, or neuropsychiatric manifestations [32–34].

In this study, we examined the carotid arterial intima-media thickness and the brain MRI measurements in SSc patients and compared them to normal controls.

There was a statistically significant difference between the SSc patients and controls for multiple laboratory parameters such as the CRP, ESR, lipid profile, ECG abnormalities, right and left CCA intima-media thickness, and lesions detected by brain FLAIR-MRI, similar to previous studies [35–37]. These differences could be explained as a result of widespread vasculopathy and extracellular matrix deposition with fibrosis and autoimmune processes as part of the characteristic pathogenesis of SSc.

There were statistically significant differences between SSc and control groups with respect to hyperintense lesions on FLAIR, involving the white matter of both cerebral hemispheres. This result agrees with those of others [33, 37, 38]. The SSc patients’ MRI with positive FLAIR lesions might represent some CNS small vessel disease, including increased ASCVD, since the lesions correlated with increased CMT.

These lesions may be the result of endothelial and basement membrane damage with platelet aggregation and a non-inflammatory micro-angiopathy of the vasa nervorum, ultimately leading to ischemic injury, another theory postulates a non-immune reaction with cell-mediated activation and secondary autoantibody production. Our data support either or both of these pathogenetic models as we found the final result of such mechanism consisting of a thickened carotid intima-media complex. A third pathway with alterations of collagen metabolism, proliferation of specific fibroblast subpopulations, and tissue invasion and fibrosis may be less likely because there is a paucity of connective tissue in the CNS [38].

Although other studies found a correlation between CRP, increased CCA-IMT, and stroke, our study found only a trend in this direction (not statistically significant) [39–41]. The elevated CRP, in part, reflects the burden of atherosclerosis, but it may also reflect other factors, as it remained an independent risk factor for ischemic stroke. Further study of the interrelations of inflammation markers and subclinical atherosclerosis in relation to clinical outcomes is needed.

In our study, there was a significant difference between the SSc patients with a positive MRI and those with negative MRI findings with respect to disease duration and bilateral CCA-IMT atheromata. Speculatively, this could lead to considering a CNS MRI in SSc patients with an increased CCA-IMT. Moroni et al., in a meta-analysis, concluded that carotid atherosclerosis is a marker for susceptibility to ischemic cerebral damage, supporting this speculation [42]. Also, the SSc patients with MRIs with positive FLAIR lesions might represent some CNS small vessel disease, including possible ASCVD.

We found that there was a statistically significant correlation between the CCA-IMT and Medsger kidney domain. This is generally logical as both the carotid vessels and renal vessels may suffer from similar endothelial insults in SSc. Tyrrell et al., in a meta-analysis, found a relationship between various rheumatic diseases and carotid intima-media thickness, with the largest effect size in SSc, supporting our findings [43].

Our study had significant strengths in our careful examination of CNS MRI, carotid abnormalities, and correlation with clinical variables. However, there were also limitations. The controls were, by definition, normal and without underlying disease. We used this control group to ascertain the degree that SSc patients were different from normal. An additional control group with ASCVD would have been desirable.

Our normal control group size was not equal to the number of SSc patients, and no serological testing was done in the control group. These differences were the result of financial considerations, not allowing full detailed comparisons. Our patient group was not a selected population although there was less Raynaud’s and more scleroderma renal crisis than usual. The lack of Raynaud’s may have been secondary to the relatively warm Egyptian environment. In any case, this may affect the generalizability of the results. Our radiologist was experienced but it would have been desirable to have two independent radiographic readers. This was a cross-sectional study and a longitudinal comparison would have been of great interest. While our study group was of reasonable size, ANA positivity was relatively low (79%), while SCL-70 was positive in a high percentage (64%) and fibrosis on HRCT was lower than might be expected (33%). Further, this is a single-center study, and including multiple centers would have increased the generalizability of the results. Thus, the generalizability of our results will need corroboration.

## Conclusion

Even in systemic sclerosis patients without CNS symptoms, involvement of the CNS may occur. It may be worth considering a CNS MRI if CRP is elevated, there are abnormalities in renal function, Medsger renal scale is abnormal, or there are abnormalities in carotid intimal-medial thickness, although these data need corroboration in further studies.

## Abbreviations

ACR: American College of Rheumatology
CCA: Common carotid artery
CNC: Central nervous system
CRP: C-reactive protein
DWI: Diffusion-weighted MRI
EKG: Electrocardiogram
ESR: Erythrocyte sedimentation rate
FLAIR: Fluid-attenuated inversion recovery
FVC: Forced vital capacity
HDL: High-density lipoprotein
IMT: Intima-media thickness
LDL: Low-density lipoprotein
MRI: Magnetic resonance imaging
PASP: Pulmonary artery systolic pressure
RBS: Random blood sugar
RSS: Rodnan skin score
SSc: Systemic sclerosis
TG: Triglycerides
